# Boron Neutron Capture Therapy: Clinical Application and Research Progress

**DOI:** 10.3390/curroncol29100622

**Published:** 2022-10-18

**Authors:** Xiang Cheng, Fanfan Li, Lizhen Liang

**Affiliations:** 1 Oncology Department, The Second Affiliated Hospital of Anhui Medical University, 678 Furong Road, Hefei Economic and Technological Development Zone, Hefei 230601, China; 2 Hefei Comprehensive National Science Center, Institute of Energy, Building 9, Binhu Excellence City Phase I, 16 Huayuan Avenue, Baohe District, Hefei 230031, China

**Keywords:** boron delivery agents, boron neutron capture therapy (BNCT), thermal neutron, radiation, tumor

## Abstract

Boron neutron capture therapy (BNCT) is a binary modality that is used to treat a variety of malignancies, using neutrons to irradiate boron-10 (^10^B) nuclei that have entered tumor cells to produce highly linear energy transfer (LET) alpha particles and recoil ^7^Li nuclei (^10^B [n, α] ^7^Li). Therefore, the most important part in BNCT is to selectively deliver a large number of ^10^B to tumor cells and only a small amount to normal tissue. So far, BNCT has been used in more than 2000 cases worldwide, and the efficacy of BNCT in the treatment of head and neck cancer, malignant meningioma, melanoma and hepatocellular carcinoma has been confirmed. We collected and collated clinical studies of second-generation boron delivery agents. The combination of different drugs, the mode of administration, and the combination of multiple treatments have an important impact on patient survival. We summarized the critical issues that must be addressed, with the hope that the next generation of boron delivery agents will overcome these challenges.

## 1. Introduction

Malignant tumors are serious threats to human life and health, and radiotherapy is an indispensable part of the treatment of malignant tumors. There are many different modalities of radiation therapy, including photon radio-therapy, electron radiotherapy, proton heavy ion radiotherapy and neutron capture therapy.

Unlike other types of radiation therapy, BNCT uses cell-level short-range alpha particles for precise tumor killing. So far, there have been more than 2000 cases of clinical application of BNCT worldwide [1]. The potential of BNCT use in various disease sites, such as breast and prostate cancer, and the improved efficacy of many novel boron delivery agents are currently being extensively investigated in preclinical settings. With additional studies, BNCT has the potential to become a major means of anti-tumor therapy.

## 2. Boron Neutron Capture Therapy

Boron neutron capture therapy (BNCT) is a radiotherapy modality based on nuclear capture and fission reactions, which theoretically combines the advantages of “biological targeting” and “heavy ion radiotherapy”. The basic principle is that a boron-containing drug is injected into the patient, the boron compound accumulates in the tumor cells, and the tumor area is then irradiated with a neutron beam. After the neutron is captured by ^10^B, it transforms into ^11^B and the unstable ^11^B decays rapidly and emits α particles (^4^He) and recoil atoms ^7^Li (including ground state and excited state) in a fission reaction called ^10^B (n, α) ^7^Li fission reaction. Among them, alpha particles produced by fission are the main source of the radiation effect in BNCT.

The microscopic neutron capture cross-section of the nuclide and the nature of the fission products are two key factors in ultimately determining which element to use for neutron capture therapy (NCT). The former accounts for the ability of the nuclei used for NCT to absorb neutrons, while the latter determines the effectiveness of radiation in killing cancer cells. Referring to the data, ^10^B was found to be a qualified candidate for NCT with a neutron capture cross section of 3838 barns [2]. Neutron capture occurs when the non-radioactive component ^10^B is irradiated by low-energy (0.025 eV) thermal neutrons or high-energy (10,000 eV) epithermal neutrons, resulting in a fission reaction [3], The reaction formula is: ^10^B + n → ^7^Li + 4He; the reaction can release α particles (4He) and excited state ^7^Li cores (6.3%, total energy of 2.79 MeV) or ground state ^7^Li cores (93.7%, total energy of 2.31 MeV). A 0.48 MeV gamma photon is generated in the process of returning from the excited state to the ground state. The fission-produced alpha particle (^4^He) is a high LET particle with an LET ranging from 50 to 230 keV/μm [4,5,6]. ^4^He can induce DNA double-strand breaks, which usually lead to cell cycle arrest, followed by mitotic cell death, apoptosis or necrosis, effectively eliminating the tumor cells [7]. Furthermore, the distance that alpha particles travel in tissue is typically 50 to 100 microns, about the diameter of a cell [8,9,10]. If boron-containing drugs can be effectively enriched in tumor cells, BNCT can generate a large radiation gradient between the tumor and normal tissue. When BNCT eliminates tumor cells, it does not damage the surrounding normal tissue. Radiation-induced damage to normal tissue can be controlled at safe dose levels [11,12,13].

BNCT requires that boron-containing drugs selectively deliver sufficient ^10^B to tumor tissue (20–50 μg ^10^B/g or 10^9 10^B atoms/cell) [14] and avoid excessive boron uptake by normal tissues to maximize radiation efficacy and protect normal tissues. Therefore, boron-containing drugs need to possess the following characteristics: high uptake by tumor tissue (the boron concentration needed is >20 µg ^10^B/g tumor tissue) and low uptake by normal tissue; concentration ratios of tumor to normal (T/N) and tumor to blood (T/B) not lower than 3; fast clearance after treatment; low systemic toxicity; etc. [15].

## 3. Neutron Beams

One of the key factors in the success of BNCT is the nature of the neutron beam. To achieve the desired therapeutic effect of BNCT, the neutron flux at the tumor site needs to reach 10^12^ neutron/cm^2^ [11].

The tissue penetration of neutron beams is far less than that of traditional X-ray photons, which results in the inability to deliver sufficient neutron flux to deep tumors in vivo. This limits the application of BNCT to a certain extent. The peak flux of thermal neutron beams first applied to BNCT is at a depth of about 2–3 cm below the skin surface, and at a depth of 10 cm, the neutron flux decreases rapidly to about one-tenth of the peak. This makes it difficult to meet the treatment requirements for deep tumors.

In the mid- to late 1990s, researchers began to increase the penetration depth of neutrons. One of the improvements was to use epithermal neutron beams with higher energy than thermal neutron beams [16]. On 13 September 1994, the U.S. Food and Drug Administration (FDA) approved a dose-escalation clinical trial of BNCT using boron delivery agent BPA-F and epithermal neutron beams in Brookhaven Medical Research Reactor (BMMR). From 1994 to 2000, several clinical trials using epithermal neutrons were carried out at BMRR (USA), MIT (USA), High Flux Reactor (Netherlands) and FiR1 (Finland) [17]; Kawabata made the first attempt in Japan using epithermal neutrons in 2003 [16]. Epithermal neutrons achieved the desired effect, and their application enabled superficial tumors within 6–8 cm of the skin surface to be included in the reach of the neutron beam [4]. This improvement also changed the previous status whereby intracranial tumors could only be irradiated with neutrons in the craniotomy state. It opened the way for the clinical application of BNCT and also provided the possibility of receiving BNCT for patients who could not undergo surgery.

Before 2012, most of the neutron sources used in BNCT were experimental neutron devices based on nuclear reactor facilities. For safety reasons, the construction site of the nuclear reactor facilities are usually located away from densely populated areas. In some studies, patients needed to be injected with boron-containing drugs in a medical institution before being transferred to the reactor facility where neutron equipment for irradiation was located. At the beginning of 2009, the world’s first accelerator-based BNCT clinical irradiation system was completed at Atomic Furnace Laboratory of Kyoto University, Japan. Cell and animal clinical experiments were carried out in 2011, and the system has been used for clinical treatment since 2012. By the end of the November 2014, 510 clinical exposures had been performed using the reactor-based system [14]. Around 2014, Sumitomo Heavy Industries (Japan), Hitachi (Japan), Mitsubishi (Japan) and Neutron Therapeutics (USA) built accelerator-based neutron sources that could be installed in hospitals and produce epithermal neutron beams. More accelerator-based BNCT devices are reported in America, Russia, Britain, Italy, Israel and Argentina.

Accelerator-based neutron source facilities have many advantages over reactors. The output of the reactor is usually not easy to control, while the accelerator can allow researchers to adjust the parameters of the neutron beam according to needs. The volume of the accelerator equipment is significantly smaller than that of a reactor, which can reduce the maintenance costs of equipment; on the other hand, the higher safety of the accelerator guarantees the possibility to be built in medical facilities. Accelerator-based devices are likely to be the inevitable trend of BNCT in the next few decades.

In addition to directly enhancing the penetration ability of neutrons, researchers are also starting from other angles to enhance the penetration depth. For example, reducing the attenuation of neutrons in tissues is also a feasible means. According to Sakurai, the penetration depth of neutrons can be increased by draining out the cerebrospinal fluid (CSF) in the tumor-removed cavity and injecting air through an Ommaya’s reservoir [18]. This method can reduce the attenuation of neutrons in the cerebrospinal fluid, which can increase the penetration depth of neutrons and improve the neutron flux of deep tumors [19]. Kawabata successfully treated several patients with malignant meningioma with this technique [20].

In addition to the development of a more penetrating neutron beam on the basis of the accelerator, in the future, neutron beams may be able to be irradiated into the cavity during minimal invasive surgery using a compact neutron-beam guiding device instead of penetrating the body. Deep-seated tumors could be treated, which could also increase the application range of BNCT.

## 4. Boron Delivery Agents

### 4.1. First-Generation Boron Delivery Agents

Neutrons were discovered in 1932 by Professor James Chadwick of Cavendish Laboratory at Cambridge University [21]. In 1936, Gordon Locher published a discussion on the biological effects and therapeutic possibilities of BNCT. He pointed out that if a sufficient amount of ^10^B accumulates in tumor cells and is exposed to thermal neutrons, the radiation dose received by tumor tissue would be much greater than that of normal tissue. This makes boron neutron capture a viable treatment for cancer [22]. Boron is the most effective atom in neutron capture therapy, but the development of boron drugs was limited by the technical conditions at that time. Only boric acid, borax and pentaborate were synthesized.

Kruger [23] and Zahl [24] reported BNCT-related animal experiments in 1940. The first clinical application of BNCT was performed at Brookhaven National Laboratory (BNL) in 1951, at the neutron facility of Brookhaven Medical Research Reactor (BMMR). However, due to the injection of the non-targeting boron compound, it could not be effectively enriched in tumor cells, resulting in a low ratio (<1) of the concentration of boron in tumor cells to that in normal cells and blood. Therefore, the killing effect on tumor cells was not ideal [25]. Several attempts have been made since then. From 1959 to 1961, several patients with intracranial tumors underwent BNCT at BMRR; during the same period, 17 patients with malignant gliomas underwent BNCT at the Massachusetts Institute of Technology (MIT) reactor [26]. The median survival time of these patients was only 87 days. These trials used different boron compounds and various surgical interventions, but the findings were unsatisfactory. Slatkin’s animal study showed that during neutron irradiation, the concentration of boric acid in the blood was three times higher than that in the brain parenchyma, that is, vascular endothelial cells absorbed more radiation from alpha particles and ^7^Li particles than brain parenchymal cells. Endothelial cell injury may be a major determinant of acute lethality in CNS radiation syndrome [27].

All clinical trials of BNCT in the United States were discontinued in 1961 based on serious adverse events. The key defect of the first-generation delivery agents represented by boronic acid was the lack of tumor targeting. Their concentrations in tumors are very low compared to normal tissue, and their accumulation in tumor cells is transient [25,27] and cannot be used as boron carrier in clinical settings.

### 4.2. Second-Generation Boron Delivery Agents

Around the 1960s, two boron compounds that later proved to be effective, boron phenylalanine (BPA) and boron card sodium (BSH), were first synthesized in 1958 and 1967, respectively. They are less toxic than first-generation boron compounds, have a longer duration of intratumoral enrichment and have a ratio of boron concentrations greater than 1 in T/N and T/B [12]. In 1968, Hatanaka began to try BNCT in Japan, using BSH as a boron delivery agent, and successfully treated many patients with high-grade glioblastoma (GBM). After long-term follow-up observation, the 5-year survival rate of his patients was 58%, and the 10-year survival rate was 29% [28].

Hatanaka’s and Soloway’s work in the field is in the spotlight [29,30]. Since the 1980s, with the improvement of neutron beams and the availability of second-generation boron compounds, especially after the successful purification of the levorotatory enantiomer of BPA (L-BPA) in 1980 [31], some countries in Europe and the United States have once again set off a research boom in BNCT. Finland conducted BNCT research using an FiR-1 reactor with the support of VTT Corporation [32]. The Coderre team in the United States used BPA to treat glioma tumor-bearing mice, and its therapeutic effect reached expectations f [33]. The birth of second-generation boron delivery agents laid the foundation for the clinical research of BNCT. So far, they have successfully treated more than 2000 patients with malignant tumors, and they are also the only two drugs that are currently allowed to be used in clinical practice.

### 4.3. Third-Generation Boron Delivery Agents

The development of boron delivery agents has ushered in a new opportunity with the improvement of synthetic techniques and the increased understanding of the biochemical properties of groups. In order to meet the needs of BNCT and broaden the clinical application of BNCT, many new boron delivery agents have emerged.

There are many types of third-generation drugs, including boronated amino acids, polypeptides [34,35,36,37], protein [3]; boronated porphyrins, folate receptors, boronated DNA intercalators, carboranyl nucleosides [38,39,40,41], Borated Epidermal Growth Factor (EGF) [42,43], boronated MAb to epidermal growth factor receptor (EGFR) [44]; Liposome Boron Delivery Agent [45,46,47], transferrin liposomes [48]; boron-containing nanomaterials [49,50], BSH polymeric micelles [51], etc. And also including the modification of BPA and BSH [15,52,53,54]. The effectiveness of BNCT depends on the intracellular localization of ^10^B [7,55].

With random distribution within the cell, it is estimated that about 10^9^ atoms of ^10^B are required to kill tumor cells. Boron delivery agents that specifically target DNA are expected to significantly reduce this amount [14]. Therefore, delivery agents that can localize boron in the nucleus, such as boronated nucleotides or their precursors, may be ideal agents for the selective delivery of ^10^B into cancer cells. This precise targeting capability is expected to reduce the dose of boron drugs required for therapy, thereby reducing potential drug toxicity and the neutron dose required for irradiation.

## 5. Application of Second-Generation Boron Delivery Agents

Clinical studies of second-generation boron delivery agents are listed in Table 1 [56,57,58,59,60,61,62,63,64,65,66,67,68,69,70,71,72,73,74,75,76,77,78,79,80,81,82,83,84,85,86,87,88,89,90,91,92]. Current research originates from many institutions around the world. There is a high degree of heterogeneity in terms of patient ethnicity, inclusion criteria, physician screening of patients, use of boron delivery agent, way of administration, neutron dose administered and care after BNCT. It is far from enough to judge the pros and cons of a treatment plan only from patient survival information, so caution should be exercised when interpreting the data in the table below.

### 5.1. Cell Uptake

Boronphenylalanine (BPA) is an analog of tyrosine (the raw material of melanin) that gives it the ability to be actively taken up by cells, especially melanoma. BPA can be transported into cells through the L-aminoacid transporter-1 (LAT-1) system, and the expression of LAT-1 is highly upregulated in a variety of tumors; it is speculated that it can promote tumor growth by increasing amino acid supply [42,93]. BPA is an amino acid derivative, and there is a certain balance in the uptake of BPA by cells. For example, the presence of phenylalanine in the nutrient medium may affect intracellular free boron; the majority of the boron pool from BPA in GBM cells can be decreased by extracellular phenylalanine and possibly by other competing amino acids [94]. Therefore, tumor cell uptake of BPA can be increased by administering a low phenylalanine diet to patients prior to BNCT or by preloading with L-tyrosine [93,94,95]. Wittig et al. studied the biodistribution of BPA in normal tissues in a mouse model, and the average concentration in the kidney reached 37.8 ± 24.8 μg/g, which is the main excretion pathway of BPA [96,97].

BSH (Sodium borocaptate ^10^B, Na2B12H11SH) is a polyhedral borane containing 12 boron atoms. In the case of similar molecular weights, its boron-carrying rate is much higher than that of BPA. BSH cannot cross the intact blood–brain barrier (BBB), so it can be safely transported in the cerebral blood vessels with little uptake by normal brain tissue [98], while in tumor regions where the blood–brain barrier is disrupted, BSH can enter tumor cells via passive transport, and its transport depends only on the vasculature of the tumor tissue. This feature allows BSH to achieve a T/N ratio much higher than that of BPA and a T/B ratio close to 1 [99,100,101]. However, since BSH can only be taken up passively, it is difficult to apply to extracranial tumors alone.

### 5.2. 18F-BPA Simplifies Concentration Monitoring

In the early 1990s, due to the lack of effective monitoring methods, the detection of ^10^B biodistribution in patients needed to be performed on the basis of surgically obtained blood and tissue [102]. This greatly limited the selection of patients who may have benefit from BNCT. Mishima et al. designed a drug, ^18^F-BPA, that can detect ^10^B distribution via positron emission tomography (PET) [103]. ^18^F-BPA is also a substrate of LAT1 and has a tumor-transporting capacity comparable to that of BPA [104,105,106,107]. Using ^18^F-BPA-PET technology not only can non-invasively (injection only) screen out the patient group who can really benefit from BNCT treatment, but it is also conducive to accurate radiation dosimetry design. In the pre-uptake analysis using ^18^F-BPA-PET, the BPA concentration at the tumor site during the implementation of BNCT can be estimated by taking blood samples instead of collecting solid tissues [108]. This technique has until now been used as one of the key means of BNCT assessment in patients [20,66,68].

### 5.3. Clinical Application of BNCT in Intracranial Tumors

**Single-drugBNCT:** Diaz et al. treated a total of 53 patients with newly diagnosed glioblastoma (GM) at BMRR between 1994 and 1999 with a 2 h infusion of 250–330 mg/kg of BPA [84]. The 1996–1999 Harvard–MIT study with similar doses and administrations treated 20 newly diagnosed GMs [85]. The median survival time (MST) after diagnosis in the two groups was 12.8 months and 11.1 months, respectively. Skold, K., used BPA infusion for up to 6 h, with a total infusion volume of 900 mg/kg body weight, and found that patients who used BPA-F for a long time had a survival advantage [109]. However, even with a 6 h infusion, the median time to progression (TTP) after BNCT was less than half a year (5.8 months). Two studies used 100 mg/kg BSH infusion at High Flux Reactor (Petten, the Netherlands) and LVR-15 Reactor (the Czech Republic); the former patients had an MST of 10.4–13.2 months after BNCT [72], and the latter patients had a TTP of less than half a year (2.5–6 months) after treatment (the latter’s MST data are not available) [83].

Although patients who received single-agent BNCT achieved a certain curative effect, the overall survival effect did not show obvious advantages, which indicated that single-agent BNCT was still insufficient in the treatment of glioma. The study found that BPA is preferentially taken up into actively proliferating tumor subsets and cannot accumulate in quiescent cells, which may be the cause of disease recurrence after BNCT due to insufficient boron uptake [110]. This is not only true for BPA, as Takagaki observed in a study that BSH was also unevenly distributed in tumors [101]. There is significant variability in tumor uptake of BPA and BSH, especially in brain tumors, where boron concentrations vary among the different regions of the tumor and among patients receiving the same dose. Heterogeneity in tumors may cause some cells to lack the ability to accumulate boron compounds to escape death and eventually relapse after BNCT. The combined use of BSH and BPA can improve the uneven distribution of boron concentration in tumors.

It has also been shown that the use of pulsed ultrasound can instantaneously disrupt the blood–brain barrier while improving the uptake of the delivery agent and promoting the microdistribution of ^10^B in intracranial gliomas, and head and neck cancer (HNC) [111,112].

**Clinical study of BPA combined with BSH:** Kawabata et al. attempted a combination of BSH and BPA and successfully treated two patients [16]. Tokushima University treated six cases of newly diagnosed GM from 2001 to 2004; the patients’ post-diagnosis MST reached 26.2 months, and the two-year survival rate was 50% [62,63,64]. Miyatake et al. conducted a clinical trial using a combination of 5 g of BSH plus 250 mg/kg of BPA in KUR, and 10 patients with GM received BNCT. It was found that 2–7 days after treatment, the tumor volume was reduced by 17.4–71% via imaging assessment. The long-term follow-up showed that the tumor volume decreased by 30.3–87.6%, and the tumor volume decreased by more than 50% in eight patients. The MST after BNCT was 14.5 months [65]. Compared with single-agent BNCT, the combination of BSH and BPA effectively prolonged the survival of patients, and there were no obvious toxic and side effects associated with the combination.

Worldwide, in clinical studies of BPA combined with BSH, patients could relapse in 1 month; in contrast, there were also cases with survival greater than 6 years [56]. The effectiveness of treatments varied widely. In 2006, Miyatake reported an interesting case of a patient with gliosarcoma who had previously received BPA–BSH dual-drug-infusion BNCT and had only relapsed sarcomatous components 6 months after treatment [113]. Even with the combined use of BSH and BPA, there may still be tumor cells that are low in boron enrichment and escape death. Tamura [114] reported a 25-year-old female patient in preparation for the second BNCT treatment; ^18^F-BPA PET found that the T/N ratio of recurrent lesions had decreased from 5.0 after the first treatment to 1.9. Since the effective killing range of α particles only reaches the cell diameter, and the heterogeneous tumor cells are distributed in the tumor mass, the high-uptake tumor cell area is more obviously destroyed in BNCT, while low-uptake tumor cells may survive. After screening using BNCT, the proportion of low uptake in residual lesions is higher than before, and this proportion may be preserved to a certain extent at recurrence, thus reducing the T/N ratio at the second BNCT session.

**BNCT combined with XRT:** Tumor heterogeneity still plays a decisive role in the recurrence process after BNCT, which requires researchers to further explore the solution of uneven enrichment. Barth et al. demonstrated in animal models that the combination of BNCT and conventional photon radiotherapy could achieve significant therapeutic effects [115]. Additional X-ray treatment (XRT) may ameliorate dose shortages in deep tumors and, by increasing the baseline dose, ameliorate underdosing due to an uneven distribution of ^10^B atoms in tumor tissue [68]. In 2009, Kawabata further improved the BNCT procedure for glioma based on Barth and previous studies, adding the application of XRT after BNCT [20,67]. Twenty-one newly diagnosed GM patients were divided into two groups for treatment. Among patients at the institution who did not receive BNCT, the median survival time was 10.3 months. Patients who received BNCT (group one) had an MST of 14.1 months. Patients receiving BNCT and XRT (group 2) achieved a post-treatment MST of 23.5 months. The additional application of XRT greatly prolonged patient survival. In Yamamoto’s study, eight newly diagnosed GM patients received BNCT followed by a total X-ray dose of 30 Gy in 15 fractions or 30.6 Gy in 17 fractions. The median survival after BNCT was 27.1 months, TTP was 11.9 months, and the 2-year survival rate was 63%. In this series, no apparent adverse effects based on BNCT were observed [60]. The combination of BNCT and XRT could achieve better efficacy than BNCT alone.

**BNCT combined with temozolomide (TMZ):** The standard treatment regimen for newly diagnosed GM is maximum tumor resection, plus concurrent temozolomide chemoradiotherapy followed by temozolomide adjuvant chemotherapy (Stupp regimen). In the Stupp study, the MST of the newly diagnosed GM patients after radiotherapy alone was 12.1 months; the MST of the patients who underwent postoperative temozolomide concurrent chemoradiotherapy and adjuvant chemotherapy was 14.6 months. Combining tumor system therapy with BNCT may become a possible means to reduce tumor recurrence and improve patient survival. In a Swedish study, patients who received temozolomide after BNCT experienced longer survival than those who received BNCT alone (17.7 months versus 11.6 months) [80]. In an unpublished study by Miyatake et al., BNCT + XRT combined with temozolomide was used to treat 32 patients with newly diagnosed GBM (Osaka-TRI BRAIN 0902, NCT00974987) [68,116,117]. In this prospective study, patients’ MST was 21.1 months, and 2-year OS was 45.5%. This shapes a new combination therapy model for BNCT.

**BNCT combined with bevacizumab:** There are high rates of pseudoprogression and brain radiation necrosis in patients with malignant glioma undergoing BNCT [118]. In a study, 11 of 52 patients with malignant glioma and 3 of 13 patients with malignant meningioma had increased edema 3 months after BNCT [119]. The occurrence of radiation necrosis depends on vascular endothelial growth factor (VEGF). The researchers tried BNCT combined with the VEGF monoclonal antibody bevacizumab. From 2013 to 2014, seven patients were enrolled [70]. Bevacizumab was started 2–6 weeks after BNCT at 10 mg/kg every two weeks. As of December 2017, no radiation brain necrosis was found in none of the enrolled cases. The results demonstrated that combined bevacizumab treatment could prevent radiation necrosis and prolong patient survival. In another study, the administration of bevacizumab at a dose of 5 mg/kg improved pseudoprogression after BNCT in two patients with recurrent glioma [120]. In four patients with recurrent malignant glioma treated with BNCT and bevacizumab in Osaka, their MST after BNCT was 14 months, 16.5 months, >23 months, and >26 months, respectively. These results support that combining BNCT with bevacizumab improved pseudoprogression or radiation necrosis and prolonged patient survival [121].

Kawabata et al. performed BNCT in 19 patients with recurrent meningioma from 2005 to 2011; each patient was given 500 mg/kg of BPA plus 5.0 g, 2.5 g or 0 g BSH. If BSH was used, compound solutions were administered in 1 h, starting 12 h prior to neutron irradiation. The injection of BPA was divided into two sections, with 200 mg/kg/h being given 2 h before neutron irradiation and 100 mg/kg/h being given during irradiation. The dose of neutron irradiation did not exceed 15 Gy-Eq for the normal brain [69]. In total, 18 of the 19 patients who underwent ^18^F-BPA-PET showed favorable BPA uptake with a T/N greater than 2.7. Patients’ mean tumor volume decreased by 64.5% within 2 months after BNCT. The patient’s post-BNCT MST was 14.1 months, and that post-diagnosis was 45.7 months. Clinical symptoms before BNCT, such as hemiplegia and facial pain, were improved after BNCT in symptomatic cases. In the clinical observation after BNCT, there were six patients with systemic metastasis, seven patients with intracranial distant recurrence in the radiation field, three patients with cerebrospinal fluid dissemination and three patients with local tumor progression. Significant pseudoprogression was observed in at least three cases. Symptomatic radiation injury occurred in six cases, and all but one were controllable [69].

### 5.4. Extracranial Tumor

Due to the limited penetration of neutrons, non-operative BNCT is only suitable for superficial tumors. In addition to gliomas and meningiomas, BNCT is also used in the treatment of melanoma, head and neck tumors, and extramammary Paget’s disease, and it also achieved good therapeutic effect. Although there are related reports on lung cancer [58] and liver cancer [122,123], the clinical evidence on the efficacy is still insufficient, and further research is needed to reveal it in the future.

BPA is an analog of tyrosine, and tyrosine is a synthetic substrate of melanin, which means that BPA may show great advantages in the treatment of melanoma. The use of BPA in the treatment of melanoma was first reported by Mishima in 1987 [124]. Between 1987 and 2001, Fukuda performed BNCT on 22 patients with malignant melanoma, with 170–200 mg/kg BPA perfusion for 3–5 h before a total of 60–90 min of irradiation. The complete remission (CR) rate after treatment was 73% (16/22); the partial response (PR) rate was 23% (5/22); the local response rate (CR + PR) reached 95%. Based on the study by Mishima [124] and Fukuda [89], Junichi, H., published a long-term observation of BNCT in melanoma. From 2003 to 2014, eight patients with cutaneous melanoma received BNCT. The age distribution was 48–86 years old, and all patients had carcinoma in situ located on the sole or face; six patients were histologically confirmed to have acral lentiginous melanoma (ALM), and two patients had lentiginous malignant melanoma (LMM).

Before BNCT, CT, MRI, inspection or palpation was used to evaluate tumor and pigmented plaque size. No regional lymph node involvement, distant metastasis or second malignancy was observed in any patients. After a long-term follow-up after treatment, the overall control rate of eight melanoma patients was as high as 88%, and only one patient relapsed, showing that BNCT could bring long-term survival benefits to melanoma patients and is a promising treatment method [125]. Since 2003, Argentina has carried out clinical phase I and phase II trials of BNCT in the treatment of melanoma, with an effective rate of about 69.2%. Seven patients were enrolled, including six females and one male, ranging in age from 51 to 74 years old. All of them had multiple subcutaneous metastases in the lower extremities/feet. After BNCT treatment, the complete remission rate of multiple subcutaneous metastases could reach from 9% to 100% [92].

There are a large number of important organs sensitive to radiation in the head and neck region. BNCT can target and kill cancer tissues while causing less damage to normal tissues. It could be an ideal solution for radiation therapy of head and neck cancer. Osaka University was the first to apply BNCT to head and neck cancer, giving BPA or BPA and BSH perfusion, and achieved an overall response rate of 85%. The 26 patients who participated in the experiment had an average survival time of 13.6 months and a 6-year survival rate of 24% [56]. Suzuki reported the records of 62 patients with locally recurrent or unresectable head and neck cancer treated with BNCT at Kyoto University between 2001 and 2007, and the patients received BPA or BPA and BSH perfusion. The patients had an MST of 10.1 months, and the overall response rate at 6 months was 58%, while the 2-year OS rate was 24.2% [57]. Kankaanranta et al. reported a prospective phase I/II trial conducted in Finland from December 2003 to September 2008 (NCT00114790). This trial performed BNCT in 30 patients with inoperable locally advanced head and neck cancer. Patients received two phases of BPA dosing, totaling 400 mg/kg. Among 29 evaluable patients, there was a 76% response rate. Median PFS was 7.5 months, and 2-year OS and PFS were 30% and 20%, respectively [126].

In 2012, Makino first reported the use of BNCT for the treatment of extramammary Paget’s disease (EMPD), and two patients had a PFS greater than 12 months [90]. In Hiratsuka’s study, three patients received a total of 200 mg/kg BPA perfusion (of which 40 mg/kg was given during neutron irradiation). One patient died of heart disease 3.2 years after treatment, and the remaining two patients survived for more than 6 years [91]. Although the total number of cases was small, BNCT proved to be a promising modality for the treatment of EMPD.

BPA and BSH have held an unparalleled position for nearly 30 years, but they still have many shortcomings. BPA is poorly water soluble and usually requires fructose as a solvent. Due to the nature of its amino acid analog, it is also taken up by normal tissues, resulting in an insufficient T/N ratio in many patients. BSH can only be passively transported into cells, and its high T/N ratio is dependent on the blood–brain barrier, making it difficult to apply to diseases outside the central nervous system. In addition, BSH has become difficult to obtain due to its high production cost [69]. After 2010, in many clinical studies on glioma and meningioma, BSH had to be reduced or discarded. This makes the development and efficacy validation of a new generation of boron delivery agents increasingly important.

## 6. Additional Considerations When Utilizing BNCT

In the process of neutron irradiation, in addition to the capture reaction of ^10^B to neutrons, elements in the human body can also interact with neutrons. The neutron cross sections of common elements in the body are shown in Table 2. Although the neutron cross section of the boron atom is much larger than that of other elements in the table, the latter are the main elements constituting the human body, and their high quantities in tissues significantly increase the incidence of neutron capture events. The macroscopic cross-section of the element can be obtained by synthesizing the neutron cross-section and the content of the element in the human body. The main reactions during neutron irradiation are: ^10^B(n, α)^7^Li, ^14^N(n, p)^14^C and ^1^H(n, γ)^2^D.

Increasing the T/N ratio of boron or the boron concentration in the tumor can reduce the neutron exposure time required for treatment. Not only does it reduce radiation exposure to normal tissues from boron uptake, it also reduces the number of constituent elements–neutron reaction events. Using two or more irradiation fields can directly reduce the dose to normal tissue.

The main sources of side effects after BNCT are: 1. Radiation effects caused by the uptake of boron drugs by normal tissues, such as hair loss, skin damage, mucositis, some neurological symptoms, etc. 2. The damage to vascular endothelium caused by boron-containing drugs in the blood. 3. Accompanying changes after tumor necrosis, such as cerebral edema.

Since boron drugs inevitably exist in normal tissues, as the radiation dose increases, the incidence of side effects also increases, which is not beneficial to the patient’s PFS and survival time [84,127]. This shows that the survival benefit of BNCT for patients is achieved under the appropriate neutron irradiation dose. Insufficient doses can lead to incomplete tumor killing, resulting in recurrence and/or decreased OS, while at doses effective to kill tumor cells, further neutron irradiation may reduce PFS and multiply damage to normal brain tissue. Therefore, a rigorous and individualized dose plan becomes one of the key factors affecting the prognosis of BNCT patients. We cannot pin our hopes for the development of BNCT on new boron delivery agents and more powerful neutron penetration. Exploring more optimized irradiation patterns or more individualized adjustment of details may also bring considerable benefits to patients, which requires more evidence and research to open up avenues.

## 7. Conclusions

In summary, BNCT is a binary system that combines the characteristics of biological targeting and radiotherapy, which can form a large dose gradient between adjacent cancer cells and normal cells, enabling it to precisely kill tumor cells. The concept of BNCT has been around for more than 80 years, and the technology has advanced in many ways. Patients’ screening has replaced surgery with ^18^F-BPA-PET, and thermal neutrons have evolved into deeper penetrating epithermal neutron beams; smaller accelerator facilities have replaced the reactors, and novel boron delivery agents are continually being updated.

The main factors restricting the development of BNCT are as follows:

1. Lack of more effective boron delivery agents. Although the preliminary clinical results of BNCT based on second-generation delivery agents are promising, there are still many problems, such as the uneven distribution of intratumoral boron, insufficient T/N and T/B ratios and difficulty in obtaining some boron delivery agents. Further research is needed to develop more selective boron compounds to improve therapeutic efficacy and reduce potential drug toxicity;
2. High construction and maintenance costs of neutron facilities. Based on the current boron delivery agents and strict patient screening, BNCT is difficult to be used as a first- or second-line treatment for tumors and can only be used as an alternative when other methods cannot be implemented, which greatly reduces the number of patients. In the face of sparse cases, the comparative construction cost and maintenance cost of BNCT hinder its entry into clinical practice. The development of new boron delivery agents to broaden the application range of BNCT may be a big boost to advance its development. In addition, there are only a handful of BNCT devices in the world, and some countries do not have equipment to carry out neutron-based research. Based on the requirements of BNCT technology for neutron devices, the difficulty in connecting and collaborating between drug researchers and neutron institutions is also an obstacle to the translation from preclinical to clinical;
3. Heterogeneity of research factors. The comparative study of BNCT becomes difficult due to the large differences in many factors among the studies in different countries in the world. More well-designed clinical studies are needed in the future to determine the role of novel boron delivery agents in BNCT clinical practice. Randomized controlled animal trails should be presented to prove effectiveness of new boron drugs or new treatment modes, giving guidance to randomized clinical trials;
4. In addition, a large number of third-generation boron delivery agents have been tested and studied at the cellular and animal levels, but no further clinical trial has been conducted. One of the reasons is the lack of convincing animal experimental data. Although some studies have achieved excellent T/N and/or T/B ratios, the tumor boron concentration does not meet the minimum standard at the cellular level or in animal models (20 μg/g). Some studies obtained sufficient enrichment concentrations but with low T/N ratios. Another problem with translation from preclinical to clinical research is that not all third-generation drugs under investigation have developed PET-adapted labeled versions. This means that clinical trials may require tissue samples to verify intratumoral concentrations and T/N ratios, and such biodistribution studies with no direct benefit to patients are difficult to conduct.

The treatment mode of BNCT is developing in the direction of multi-disciplinary cooperation and multi-field intersection. In the future, BNCT can be combined with immunotherapy and targeted drugs to improve tumor targeting. It is hoped that through the efforts of experts in various disciplines, BNCT will become one of the important means of anti-tumor treatment as soon as possible, benefiting cancer patients.

## Figures and Tables

**Table 1:** Clinical application of second-generation boron delivery agents.

Institution	Year	Tumor Type	Medicine and Administration	Clinical Outcome	Ref.
Osaka University, Japan	2001–2009	HNC 26	BSH, 0 or 5 g/body in 1 h (12 h before BNCT) BPA, 250 or 500 mg/kg in 1 h (1 h before BNCT) (Some patients received 2–3 BNCT sessions)	MST: 13.6 M (post-BNCT) 6-year OS: 24%	[56]
Kyoto University, Japan	2001–2007	HNC 62	BSH, 0 or 5 g/body in 1 h (12 h before BNCT) BPA, 250 or 500 mg/kg in 2–3 h (2 h before BNCT)	MST: 10.1 M (post-BNCT) 1-year OS: 42% 2-year OS: 24%	[57]
	2012	r Lung Cancer 01	BPA, 400 mg/kg in 2 h (2 h before BNCT) BPA, 100 mg/kg in 1 h (during BNCT) (After one month, they received the second BNCT session)	PFS: 7 M	[58]
JRR-4, University of Tsukuba, Japan	1999–2002	nGBM 05	BSH, 100 mg/kg in 1~1.5 h (IO-BNCT)	MST: 23.2 M (post-BNCT)	[59]
	1999–2002	AA 04	BSH, 100 mg/kg in 1~1.5 h (IO-BNCT)	MST: 25.9 M (post-BNCT)	[59]
	1998–2007	nGBM 07	BSH, 5 g/body in 1 h (IO-BNCT)	MST: 23.3 M (post-BNCT) TTP: 12.0 M (post-BNCT) 2-year OS: 43%	[59]
	1998–2007	nGBM 08	BSH, 5 g/body in 1 h BPA, 250 mg/kg in 1 h (BNCT and XRT)	MST: 27.1 M (post-BNCT) TTP: 11.9 M (post-BNCT) 2-year OS: 63%	[60]
	2007	HNC 01	BPA, 400 mg/kg in 2 h (2 h before BNCT) BPA, 100 mg/kg in 1 h (during BNCT) (After one month, they received the second BNCT session)	6 M after first BNCT, the tumor shrank significantly OS: NA	[61]
Tokushima University, Japan	1998–2000	nGBM 06	BSH, 100 mg/kg (12–15 h before BNCT) (IO-BNCT)	MST: 15.3 M (post-diagnosis) 2-year OS: 0%	[62,63,64]
	2001–2004	nGBM 11	BSH, 100 mg/kg (12–15 h before BNCT) (IO-BNCT)	MST: 19.5 M (post-diagnosis) 2-year OS: 27.3% 3-year OS: 27.3%	[62,63,64]
	2001–2004	nGBM 06	BSH, 100 mg/kg (12 h before BNCT) BPA, 250 mg/kg (1 h before BNCT)	MST: 26.2 M (post-diagnosis) 2-year OS: 50.0% 3-year OS: 16.7%	[62,63,64]
	2002–2003	nGBM 03 rGBM 07	BSH, 5 g/body in 1 h (12 h before BNCT) BPA, 250 mg/kg in 1 h (1 h before BNCT)	MST: 14.5 M (post-BNCT)	[65]
KURR, Osaka Medical College, Japan	2002–2007	rGBM 19	BSH, 100 mg/kg in 1 h (12 h before BNCT) BPA, 250 mg/kg in 1 h or 700 mg/kg in 6 h (6 h before BNCT)	MST: 10.8 M (post-BNCT)	[66]
	2002–2004	nGBM 10	BSH, 100 mg/kg in 1 h (12 h before BNCT) BPA, 250 mg/kg in 1 h (6 h before BNCT)	MST: 14.1 M (post-BNCT)	[20,67]
	2003–2006	nGBM 11	BSH, 100 mg/kg in 1 h (12 h before BNCT) BPA, 250 mg/kg in 1 h (6 h before BNCT) (BNCT and XRT)	MST: 23.5 M (post-BNCT)	[20,67]
	2010–2013	nGBM 32	BSH, 5 g/body in 1 h (12 h before BNCT) BPA, 500 mg/kg in 3 h (2 h before BNCT) (BNCT, XRT and TMZ)	MST: 21.1 M (post-BNCT) (2-year OS: 45.5%)	[68]
	2005–2011	rMM19	BSH, 0 or 2.5 or 5.0 g/body in 1 h (12 h before BNCT) BPA, 400 mg/kg in 2 h (2 h before BNCT) BPA, 100 mg/kg in 1 h (during BNCT)	MST: 14.1 M (post-BNCT) MST: 45.7 M (post-diagnosis)	[69]
	2013–2018	rGBM 10	BPA, 400 mg/kg in 2 h (2 h before BNCT) BPA, 100 mg/kg in 1 h (during BNCT) (BNCT and Bevacizumab)	MST: 12 M (post-BNCT) TTP: 5.4 M (post-BNCT)	[68,70]
	2016–2018	rGBM 24	SPM-011, 400 mg/kg in 2 h (2 h before BNCT) SPM-011, 100 mg/kg in 1 h (during BNCT)	MST: 18.9 M (post-BNCT) 1-year OS: 79.2%	[71]
High Flux Reactor, Netherlands	1997–2002	nGBM 26	BSA, 100 mg/kg in 1.7 h	MST: 10.4–13.2 M	[68,72]
FiR-1 or Triga Mark II Reactor, Finland	1999–2001	nGBM 18	BPA, 290~400 mg/kg in 2 h (irradiation started 45 min after injection)	6 MOS: 100% (post-BNCT) 1-year OS: 61% (post-BNCT)	[73]
	1999–2001	rGBM 03	BPA, 290 mg/kg in 2 h (irradiation started 45 min after injection)	OS: 5 M, 7 M, >12 M (post-BNCT)	[73]
	2001–2008	rGBM 20 AA 02	BPA, 290~450 mg/kg in 2 h (2 h before BNCT)	MST: 7 M (post-BNCT) TTP: 3 M (post-BNCT) 1-year OS: 36%	[74]
	2003–2012	rHNC 79	BPA, 350~400 mg/kg in 2 h (2 h before BNCT) (39 patients received a second BNCT session)	MST: 10 M (post-BNCT) 2-year OS: 21%	[75,76,77]
Nyköping hospital, Sweden, R2-0 reactor	2001–2002	nGBM 17	BPA, 900 mg/kg in 6 h (8–9 h before BNCT)	MST: 12.4 M (post-BNCT)	[78]
	2001–2003	nGBM 29	BPA, 900 mg/kg in 6 h (8 h before BNCT) (13 patients received BNCT and TMZ)	TTP (total): 5.8 M (post-BNCT) MST (total): 14.2 M (post-BNCT) MST (B): 11.6 M (post-BNCT) MST (B + T): 17.7 M (post-BNCT)	[79,80]
	2001–2005	rGBM 12	BPA, 900 mg/kg in 6 h (8 h before BNCT)	MST: 8.7 M (post-BNCT) MST: 22.2 M (post-diagnosis) TTP: 6 M (post-BNCT, 11 cases)	[81]
	2003–2004	Case1: rMM Case2: rSC	BPA, 900 mg/kg in 6 h (8 h before BNCT)	Case 1, PFS: 22 M (post-BNCT) OS: 32 M (post-BNCT) Case 2, PFS: 6 M (post-BNCT) OS: >26 M (post-BNCT)	[82]
LVR-15 Reactor, Czech Republic	2000–2002	nGBM 05	BSH, 100 mg/kg in 1 h	TTP: 2.5–6 M (4 cases) (One patient died of pulmonary embolism in the first week after irradiation)	[83]
BMRR, U.S.	1994–1999	nGBM 53	BPA, 250~330 mg/kg in 2 h (irradiation started 45 min after injection)	TTP: 18–34.5 W (post-diagnosis) MST: 12.8 M (post-diagnosis) 2-year OS: 9.4%	[84]
Harvard and MIT, U.S.	1996–1999	nGBM 20	BPA, 250–350 mg/kg in 1~1.5 h (irradiation started 45 min after injection)	MST: 11.1 M	[3,85,86]
	2002–2003	nGBM 06	BPA, 14 g/m^2^ in 1.5 h (irradiation started 45 min after injection)	NA	[87]
THOR, National Tsing Hua University, Taiwan, China	2010–2013	rHNC 17	BPA, 360 mg/kg in 2 h (2 h before BNCT) BPA, 45 mg/kg in 30 min (during BNCT) (15 patients received a second BNCT session)	2-year local area control rate: 28% 2-year OS: 47%	[88]
	2014–2018	rHNC 07	BPA, 360 mg/kg in 2 h (2 h before BNCT) BPA, 45 mg/kg in 30 min (during BNCT) (BNCT and IG-IMRT)	1-year OS: 56%	[88]
KURR, Japan	1987–2001	MM 22	BPA, 170–200 mg/kg in 3~5 h	CR: 73% (16/22) PR: 23% (5/22)	[89]
	2012	EMPD 02	NA	PFS: >12 M	[90]
	2005–2014	MM 01 EMPD 03	BPA, 160 mg/kg in 2 h (2 h before BNCT) BPA, 40 mg/kg in 1 h (during BNCT)	OS (Melanoma): 1.1 years OS (EMPD, 2 cases): >6 years (One EMPD patient died of heart disease after 3.2 years)	[91]
RA-6, Argentina	2003–2007	MM 07	BPA, 14 g/m^2^ in 1.5 h (1 patient received a second BNCT session)	ORR: 69.3% CR: 9–100%	[92]

HNC, head and neck cancer; GBM, glioblastoma; AA, Anaplastic Astroglioma; MM, meningioma; SC, Stromal Chondrosarcoma; EMPD, extramammary Paget’s disease; IO-BNCT, intraoperative BNCT; n, newly diagnosed; r, recurrent; NA, not available; TMZ, temozolomide; XRT, X-ray treatment; n-year OS, n-year overall survival rate.

**Table 2:** Neutron capture cross sections of common elements.

Element	Neutron Cross Sections (Barns)
^16^O	0.0002
^12^C	0.0037
^1^H	0.332
^40^Ca	0.44
^22^Na	0.536
^14^N	1.75
^40^K	2.07
...	...
^10^B	3836

## Data Availability

All data generated or analyzed during this study are included in this published article.
